# Indents

**Published:** 1905-07-15

**Authors:** 


### INDENTS.

**☞ Brief Articles of Technical or Scientific Value will receive notice and comment. Contributions invited.**

**Obscene Dentistry.** The Brooklyn, N. Y., Alderman have decided that extracting teeth in a band wagon is obscene and offensive to chastity, delicacy and decency.

**Coloring Impressions.** Some one has suggested the use of a little blueing in the water for mixing the plaster for running the case, in order to facilitate the separation. This is an excellent idea, and should be used by all who have not tried it.—*Dental Hints*.

**Treatment for a Pulpless Sore Tooth.** Equal parts of oil of cloves and beechwood creosote, and enough of zinc oxid to make a paste. Use cotton fiber (just enough to convey it to the canals). A few treatments will cure almost any case.—A. E. W. in *Brief*.

**Increase of Professioal Men.** In twenty years, 1880 to 1900, engineers of all kinds increased 1,037.54 percent.; literary people, 507.87 percent; journalists, 144.05 percent; dentists 140.90 percent; lawyers, 78.63 percent; clergymen, 73.24 percent; physicians, 55.30 percent.

**Sterilizing Fluid for Dental Instruments.** Dr. J. Leon Williams, in the May *Cosmos*, gives the following solution for sterilizing instruments without rusting them. Make a saturated solution of borax in a twenty percent solution of formalin. It is practically instantaneous in its action as a germicide and instruments may be left for weeks in it without rusting.

**Quick Investment Material.** For quick investment for soldering bands clasps and teeth, the following mixtures may be kept on hand ready prepared: Equal parts of prepared chalk and fine sand, kneaded in glycering, making a plastic mass similar to mouldine.—*Dental Hints.*

**Toothache Cure.**

| | | |
|---|---|---|
| R | Carbolic Acid | ʒj |
| | Oil of Sassafras | |
| | Eugenol | aa ʒij |
| | Cocain | grs jv |

Mix and apply to carious tooth with a pledget of cotton saturated and and warmed.—E. G. McAbrey, in *Review.*

**To Prevent Sweating of the Hands.** Use equal parts of cologne and belle-donna tincture. Used two or three days will generally control excessive sweating. If systemic symptoms of belladonna appear, stop using. Dryness of the throat and dilated pupils are the first symptoms manifested.—Dr. Broomell, in *Brief.*

**Bridge Repair.** When the pins are intact as usually occurs, wind thin platinum in a figure eight about the pins and solder to a base of thin platinum. Punch holes through the base of the pins and replace in the mouth and burnish to fit the base, and crimp the barrels about the pins. Remove and bake porcelain to reproduce the facing and cement to place.—*Western Journal.*

**Mystery Solved.** The woman found dead on Culter Mountain, Colorado, last December, has been identified by her mother as Mrs. Bessie Bouton, of Syracuse, N. Y. The mother identified her by her teeth and by a scar on one of her fingers. The woman when found was nude and had been shot through the head. An attempt to burn the body with gasoline had so disfigured the face as to prevent recognition.—*American Journal.*

**Local Obtundent.** Melt together in a test tube equal parts of menthol and cocain, and add an equal amount of carbolic acid. Keep in a well-stoppered bottle. Before applying the solution to the dentine, wash the cavity with a warm alkaline solution and dry with alcohol and warm air. The lotion may also be used agreeably when scaling teeth or fitting crowns and bands.—*Dental Era.*

**Trifacial Neuralgia.** The pain of trifacial neuralgia is immediately relieved by injections of osmic acid, and in a large percentage of cases for a long period of time. All of the nerve branches should be injected, the palatine, lingual, mandibular infra and supra-orbital; they can all be exposed through small mouth incisions except the supra-orbital. Occasionally it is necessary to inject the auricular branch.—*Journal American Medical Association.*

**Retention of Rubber Dam.** Instead of using ligatures or clamps to retain the rubber close to the gum, use rubber strips, such as are used for separating teeth. After placing the dam, stretch the rubber strip and draw it between the teeth and when allowed to contract it will crowd the dam down to place and securely retain it, preventing saliva from seeping under the margins. If the rubber strips are large enough they also act as wedges to separate the teeth.—*Cosmos.*

**Deciduous Dentition Defective.** Delayed or defective deciduous dentition is generally due to a defficiency of proteid food. Bottle-fed babies, who have been given for a considerable period the cereal or patent foods, or condensed milk in which case sugar predominates, are likely to exhibit evidence of mal-nutrition in the development and eruption of the deciduous teeth. And unless the diet is changed the trouble will be manifest in the second dentition. It is simply a condition of proteid starvation.—Dr. A. B. Spach, in June *Review.*

**Lower Artificial Teeth.** Don't make them too short as the patient will protrude the jaw unnaturally and can't masticate properly or rest the jaws comfortably. It isn't always necessary to set the lower molars directly over the ride, or to set them inside to get occlusion with the upper teeth. This interferes with the free use of the tongue and spoils the speech. The teeth can, if necessary, be set out toward the buccal tissues and get a good occlusion.—Dr. Haskell, in *The Brief*.

**Gutta-percha for Setting Bridges.** Use gutta-percha warmed on the dowels and in the crowns to set the bridge leaving the canals and stump moist. Before the gutta-percha gets cold remove the bridge and cut the gutta-percha out of the crowns, but leave it on the dowels. Then dry the roots and stumps and fill the crowns of the bridge with cement, warm the gutta-percha on the dowels and force the bridge to its place and retain under pressure until the cement has set.—H. J. Goslee.

**Death from Tooth Infection.** Dr. H. A. Downing, of Cincinnati, died recently from blood poisoning caused by scratch on his finger made by a sharp point on a tooth. He suffered much pain and was unconscious for several weeks. He had practiced dentistry in Cincinnati for thirty years. —Isabelle Martin, of West Hoboken, N. Y., died recently after an illness of two weeks from septicemia caused by an an abscessed tooth. The tooth was extracted and the jaw found to be badly necrosed. Two surgical operations were made, but failed to relieve the conditions.

**Hardening Devitalized Pulps.** Dr. J. I. Hart, in the May *Cosmos*, gives his method of cleansing pulp canals after devitalizing the pulp with arsenic. Apply arsenic fiber to the exposed but not inflamed pulp for either twenty-four or forty-eight hours. With antiseptic precautions remove the bulbous portion of the pulp and place over the excised pulp remnant a pledget of cotton saturated with four percent formalin. In three days remove the dressing and extract the pulp remnants from the canals with the pliers. They will come out as tough string in one piece.

**Oral Hygiene.** When I tell you that, in my opinion, the cleanest, the easiest, the safest, the simplest and the cheapest method of keeping the oral cavity clean, preserving the integrity of the gum tissue, removing inflammation of all the buccal surfaces and the mucous membrane lining them, is by the use of bi-carbonate of soda alone, I tell you a fact that is worth more than all the prepared antiseptics that are sold in the market. Use as a wash in the strength of a teaspoonfull of bi-carbonate of soda to an ordinary glass of tepid water.—Dr. Benj. Luckey, *Items of Interest.*

**Pain from Arsenic.** Why is it that we hear so much complaint of pain when the pulp has to be devitalized? If properly handled this is a perfectly painless operation. Arsenic should not be applied to an inflamed pulp. If aching, clear away the dentine and apply pure carbolic acid on pledget of cotton, then flow over this thin cement. After a day or two remove and repeat the process, except add to the cotton a very small quantity of an arsenic preparation. This application may be necessary two or three times, but the pulp will be destroyed without pain—at least this is absolutely true in my experience.—*Hints.*

**Calcium Sulphite.** Dr. George W. Clapp, of Ottawa, Ohio, says he finds this remedy of great value in the treatment, systemically, of peridental congestion in the apical region. He administers one grain every hour until the pain ceases and the congestion subsides. The actions seems to be to deplete the congisted area, and produces a slight laxative effect on the bowels. It is a mild antiseptic and is used to correct flatulence, diarrhoea and certain forms of dispepsia. The dose varies from one-fifth to five grains. Its action in reducing peridental congestion is due to its sedative effect on the blood pressure and the depleting influence caused by its bowel action.

**Peculiarities of Edentulous Jaws.** Dr. Haskell says in the June *Review* that 95 percent have a peculiar depression over the cuspid eminence on the left side; in a majority the alveolus is shorter on the left side below the cuspid eminence; in 98 percent the tuberosity is lower on the left side, sometimes giving no room for the second molar. In many mouths the lip rises higher on the left side in talking or laughing; in many cases the natural teeth of the lower jaw are longer on the right side; the left side of the lower jaw often diverges further from the median line than the right; there is usually more absorption of the process on the left side of the lower jaw.

**Immediate Root Filling.** If there is an apical abscess and a fistulous opening through the process and gum, fit a gutta percha filling into the cavity of the crown and root, leaving a hole through it for the point of a syringe. Inject with pressure a disinfectant solution through the root and fistula. Dry out the root and fill with the following preparation on a rope of cotton: Peruvian bark and iodoform equal parts, and made into a paste with glycerine. After packing the dressing in the root, use wax or unvulcanized rubber to make pressure and force liquid out of the root and through the fistula: remove the wax or rubber and fill the cavity with cement and paint gum with iodine.—Dr. Broomell, in *Brief*.

**The Lower Plate.** Sixty years of experience leads me to believe that there is more absorption of the alveolus and jaw than formerly. It may be due to the prevailing disease pyorrhea alveolaris, but any rate it seems more common than formerly, to meet cases with absolutely no alveolar ridge on the lower jaw. In many cases it seems impracticable to make a plate that can be worn successfully. I have tried heavy metal and weighted plates, and it seems that the rubber plate after all is worn with about as much comfort as any other. I am of the opinion that the lower teeth should be saved when at all practicable, and as long as they can be so as to delay the absorption process as long as possible.—Dr. Haskell, in *Brief*.

**Anchoring Large Gold Filling.** Though it has been used before, as a principle, a new thing is the filling of the first third of occlusal, buccal and even approximal cavities (if deep) with cement, and while it is yet soft, forcing a large pellet of well-annealed gold into the mass with a large serated plugger. Cut away the surplus cement so that none will reach the margin, and then burnish back the edges after the cement has set, build up with annealed gold, hand pressure, until near the surface, then use the mallet. Dr. W. I. Brigham, of Massachusetts, gave a very fine clinic of this method, saying that often times the same retention that inlays called for was all he made, and if one's filling is thoroughly anchored in cement, it surely is as safe as an inlay, for your margins can be even better protected.—H. G. Atwater, in *Pacific Gazette*.

**Care of the Teeth.** The care of the teeth of the poor naturally begins with teaching them prophylaxis, and they must be made to understand that they can prevent some diseases of the mouth and lessen caries of the teeth. In order that the greatest benefit may be derived from dental hygiene, it should be instilled into their mind somewhat as the A B C of rudimentary education—going over and over the subject until it is comprehended. Talking alone will not suffice; it should be demonstrated in our public schools by trained teachers or scientists, who should exhibit a good form of tooth brush and show the children how to use it; this demonstration should also include the use of toothpicks, floss in its several forms as found in the market, and narrow strips cut from rubber dam, which is much better and cheaper than rubber bands.—Dr. Ambler, in *Cosmos*.

**Living to One's Self.** Dr. C. N. Johnson in his presidential address to the Illinois Dental Society, said: "If we live much to ourselves we inevitably incline to a narrow view of life and its possibilities and when egotistically disposed, we are certain to magnify our own importance and place too much significance on our own personal point of view. It is only when we encounter the opinions of others as expressed in the dignity of debate or the freedom of social intercourse, that we are brought to realize the significance of man's true relationship to his fellowmen, or our own proper normal status in the world. We need the friction of the world to round off the corners of personal peculiarity to make of us a finished human product. Association will not make men think alike, but it will destroy weak thoughts and develop strong ideals.—June *Review*.

**Haig's Mixed Diet.** Experience has conclusively demonstrated that a mixed diet of both animal and vegetable food is best suited to human digestion and assimilation. If it is desirable to avoid meat the following diet has been found a good substitute: Ten ounces of bread, two ounces of oatmeal, two to three pints of milk, two and one-half ounces of cheese, one ounce of nuts, two ounces of butter and eighteen ounces of fruits and vegetables. Such a diet ought to yield 2,500 to 2,800 calories, or units, of heat and is nourishing. Patients have lived and thrived on such a diet for years, as it contains proper portions of both animal and vegetable foods, which are both digestible and will be assimilated. It is recommended for people who suffer from headaches and chronic rheumatism. Such people find it amply sufficient to maintain a vigorous body. —Dr. Spach, in June *Review*.

**Obtaining Contact and Occlusal Surfaces in Large Porcelain Inlays.** Fill the matrix to nearly occlusion and contact in the ordinary way, being careful not to have any cracks in the inlay margins. Place the inlay in the tooth cavity and get a mash bite with modeling compound, including the adjoining tooth on both sides of the inlay. Remove the inlay from the tooth and flow a slight film of hot wax over its cavity surface and put in its proper place in the modeling compound impression. Then pour the two sides of the modeling compound impression with plaster and attach to an articulator. Remove the modeling compound when the plaster has set and the occlusal relations will be seen in the plaster models. The inlay can then be removed by chilling the wax with cold water to harden it, and the porcelain inlay can be built up to an exact occlusion and given proper contour before it is tried in the mouth.—F. E. Cheesman, in *Review*.

**Pyorrhoea Treatment.** First thoroughly scale the teeth, and then wash out the pocket with hot antiseptic solution, use plenty of the irrigant and as warm as practicable and enough force to dislodge all debris of an infectious and extraneous character. Make a solution of cocain in chloroform, about a 10 percent solution with a few drops of oil of cloves. Apply the cocain solution to the pockets and then thoroughly curet the dead tissues, being careful to thoroughly cleanse the roots and remove all necrotic tissue. After thorough irrigation, apply a 10 percent solution of sulphuric acid, by means of cotton on platinum broach to every part of the pocket. Then let the case rest three days and again irrigate with hot water and apply a 10 percent solution of tri-chloracetic acid and three days later a 10 percent solution of nitrate of silver after thorough irrigation. From this on it is simply a matter of keeping the teeth and the mouth clean and in a sanitary condition. Be careful in using the nitrate of silver as it will discolor the teeth.—Dr. L. G. Mitchell, in *Western Journal.*

**Porcelain Inlays.** I have learned that a great many dentists have not attempted to do porcelain inlay work because of the supposed difficulties lying in the way, and a doubt that they can overcome them.

Others living in the country districts have not taken it up because they cannot command the electric current as we can in the cities.

It is especially to encourage these two classes to attempt it that I write. I am not an expert in this work, I have not done a great deal of it, but what I have done has given me more satisfaction than any other part of my work, and, I may add, parenthetically, it has pleased my patients also in like manner; because there is more chance for art in it; there is also more real pleasure in it than in any other part of our work.

Not all can do it equally well; neither can all insert a gold filling equally well. Yet we do not hesitate to try, and we all improve through this practice. We can do the same with inlay work.—Dr. J. D. Moody, in *Pacific Gazette.*

**Illinois Dental Law.** The State of Illinois has enacted a new dental law and it goes into effect July 1st. The main features are that any one wishing to practice in that state must pass the examination of the State Board. Only graduates of reputable dental and medical colleges and persons who have been legal practitioners of some other State for five years just previous to locating in Illinois can take the examination. A fee of twenty-five dollars is charged for the examination and license. A rather unique clause authorizes the Board to make rules or regulations to establish a uniform and reasonable standard of educational requirements to be observed by colleges, compliance with these rules will constitute reputability. Another unusual clause empowers the Board to refuse a license or revoke a license already granted to any person who has used unfair or dishonorable means to get business or profit from conducting a dishonorable business. The law is not as exacting as many of the newer laws, but is perhaps all that a state, having so many dentists of all grades as Illinois, could get through its legislature. It is said strong pressure was made to have the Governor veto it.

**Prophylaxis and Technics.** Among all the various things which attract the attention of the practitioner of dentistry as he examines and operates on the teeth of one patient after another, there are two which seem to us to stand apart from the others and demand especial attention. They are the comparatively small number of patients who thoroughly *clean* their teeth, and the still smaller number of really well performed and well finished operations to be found in a year's experience in an average practice.

The first of these conditions should be met by the operator in accordance with the age, intelligence and social position of his patient, but in no case should it be overlooked or condoned or treated as a trivial affair.

So much of the health and comfort of the patient, and success of dental operations depends upon oral prophylaxis, that it is inexcusable in any operator to neglect the instruction of his patrons in this essential part of the service he renders them.

The chance of offending a patient should not deter the operator from demonstrating, with the aid of hand and mouth mirrors, and floss silk and cotton pledget, the fact that molars and bicuspids are often sadly neglected, even in mouths where the anterior teeth receive fair attention, and should the silk or cotton in some *unaccountable* manner be held for a second beneath the nose of the patient, the demonstration is apt to assume a very acceptable degree of interest and effectiveness.

A practical demonstration of the proper use of the tooth brush may be required in some instances, can do no harm in any case, and will quite often prove of benefit to the demonstrator himself.

With the second condition mentioned there are raised for consideration two distinct questions bearing upon its existence. The first relates to the operator himself; the second concerns a part of the education of dental students.

We feel certain that every operator knows theoretically what constitutes a perfect operation, whether it be the treatment of a septic pulp canal, the proper placing of an inlay or the correct condensation and contouring of a gold filling, and having this theoretical knowledge it remains with himself and his conscience to live up to, or away from, established ideals.

Aside from theoretical knowledge, skill, time and remuneration are the vital elements of the question, and each one must decide for himself whether he will acquire the first, afford the second and receive the third. It is not to be expected that all operators will be equally skillful, for all are not born with a genius for mechanical pursuits or an appreciation of what is beautiful and artistic, and so long as dental students are not selected in accordance with their natural proclivities, it should be an imperative part of dental education to equalize as far as possible the dissimilar personal attributes, so far as they apply to dentistry, of the members of each class.

To this end it seems to us the course in operative and prosthetic technic should be improved and amplified, and receive more time, from both instructors and students, than is now accorded it.

The increasing popularity of clinical instruction and demonstration is good evidence of its effectiveness, and we believe it would be well if each member of a class of students were required to demonstrate before his fellows, several times in each year of his course of study, some procedure in operative or prosthetic technic.

When dentists universally demand that their patients shall keep their teeth clean, and teach them how to do so, and when the purely theoretical in dental education is reduced to a minimum and the intensely practical to a maximum, we may hope for a steady increase in the number of those who are noted for their ability to perform first-class dental operations.—Dr. F. L. Platt, in *Pacific Gazette.*

